# The Structure and Measurement of Labor Value in China Based on a Sample of Children Aged 8–12 Years

**DOI:** 10.3389/fpsyg.2020.580308

**Published:** 2020-11-09

**Authors:** Xiaomei Chao, Weihua Wang

**Affiliations:** Department of Education, Hunan Normal University, Changsha, China

**Keywords:** labor values, scale development, scale validation, China, children

## Abstract

The core of labor education is to shape labor values. For China, a socialist country, the purpose of labor education is to establish labor values based on the Sinicization of Marxism. Thus, according to the theoretical analysis of labor values and the interpretation of government policies of Chinese scholars, the present study constructed a scale on labor values from five dimensions: honest labor value, equality status of labor value, cherishing labor value, loving labor value, and distribution value according to work. By using exploratory factor analysis and confirmatory factor analysis, this scale was able to achieve high reliability and validity, as well as high external correlation validity. Notably, this was the first study to develop a scale on labor values from the perspective of empirical research, promoting an empirical trend in labor value research.

## Introduction

This study constructed a scale on labor values based on Chinese fundamental realities and policies in a novel sample of high-grade primary school students. The three phases and nine steps developed by Boateng et al. (2018) as best practice in the development and validation of a scale were used. The outcome of this study is a valid and reliable multidimensional scale on Chinese labor values, which is ready for adaptation in different populations.

### Domain Identification

Labor is the logical starting point that was used in Marxism to analyze the development of human history. In the system of dialectical materialism, the concept of labor prosecutes to the end. With regard to the status of labor, Marxism’s arguments can be generalized into three ideas: labor creates the world, labor creates the history, and labor creates humanity itself (Hu and Tan, 2018). Based on the status of labor itself, labor education is particularly important. Labor education refers to a kind of educational activity involving human nature, which promotes social progress and human happiness (Chen and Liu, 2017), and it is the basis of any education activity. The nature of labor education is to cultivate correct labor values (Hu and Tan, 2018), which are determined by the choice and cognition that laborers show in the labor process (Yu and He, 2014). As man’s basic view and attitude toward labor, labor values determine the value judgment and behavioral orientation of labor (Hao, 2014). Up until now, there have been many theoretical discussions, yet almost no empirical research, about labor value’s connotations and extensions. Thus, this study intended to develop an evaluation tool for scientific quantitative assessment of labor values based on predecessors’ theoretical discussions about labor values.

At present, though there are few measuring tools in the academic world, some tools can measure similar concepts that are derived from labor values, especially work values. According to the classical definition, work value is the standard used to judge behavior and goals about work in specific occupational areas and forms the basis for an individual’s career choice (Super, 1970). Some researchers believed that work values could stably predict or explain internalized traits, behavioral expressions (Sortheix et al., 2015), or job motivations (Jambrak et al., 2014) in corresponding work environments. In terms of the content of work values’ measurement, many studies have made work values scales from different angles, including the Work Values Inventory (Super and Crites, 1957), the Minnesota Importance Questionnaire (Rounds et al., 1981), the Work Importance Profile (McCloy et al., 1999), and the Work Values Assembly (Sung et al., 2017). Throughout these scales’ contents, they mainly focused on specific work domains’ individual material and spiritual needs or work fit, such as economic rewards, occupation status, prestige, personal growth, and attitudes and behavioral patterns in the face of difficulty at work. According to the contents, work values emphasize the attitudes and behavior dispositions that are revealed in the course of work, and according to the definition, labor values are the abstracts of all subjective assessments to labor values (Hao, 2014; Tan, 2019a,b), which means that the covering range for labor values is greater than for work values. Labor values also stress laboring cognition in general and universal human values, forming the basis for work-labor values. Thus, it is necessary to further construct the contents of labor values measurement by summarizing previous theoretical analyses about the contents and purposes of labor education.

### Define Each Dimension

Values have typical regional and cultural characteristics. Previous studies have indicated that work values, which are derived from labor values, have obvious cultural differences (Robinson and Betz, 2008; Hartung et al., 2010). In the same way, labor values are also largely regional. China is a socialist country, and its value system takes sinicized Marxism as the theoretical basis of its guiding ideology (Chang, 2010). Accordingly, the emphasis of this study is to construct labor values based on a summary of relevant theoretical research by typical Chinese scholars. Tan (2019b) believed that in the context of labor values, labor education should address two points: one is that we should establish correct labor concepts and positive labor attitudes, rejecting negative values such as indolence and profit at others’ expense; the other point is to cultivate the value attitude of respecting and enjoying the labor process, fruits of labor, and labor body. The formulation “Profit by other people’s toil” highlighted the significance of distributing values according to work; the respect for fruits of labor highlighted the values of treasuring fruits of labor; and the respect of different labor bodies highlighted the non-discriminatory labor value that everyone is equal before labor; at the same time, the loving labor process highlighted the values of loving labor. Liu (2019) holistically explored connotations of labor education from different angles. In particular, he proposed an idea that productive labor education is the most fundamental and necessary base for the establishment of correct labor values. Meanwhile, he expounded functions of productive labor education on cultivating labor values in order to impress upon students that labor is hard-worn, that labor is greatness, and that laborers and the fruits of labor are worthy of respect. These formulations referred to values of treasuring labor, loving labor, and respecting different labor. Moreover, more definitely, he proposed that instilling and developing labor education systems based on Chinese socialism are necessary for the construction of a culture of respecting labor, loving labor, and advocating for labor. In addition to scholastic research, official statements are also important in China. Serious attention to labor education issues roots in important leaders’ speeches or in policy promotion. In China, there have been many researchers who have sorted out the political dimension’s systematic promotion of labor education and labor values from different angles (Li and Qu, 2018; Zhou, 2019). After China entered the period of Jinping Xi, Chairman Xi repeatedly expounded issues about labor education and values; for example, in 2013, Chairman Xi pointed out that to achieve the objective of a struggle of the party and to create a bright future, we must rely firmly on the people and always advocate for them, and we must work hard, honestly, and creatively (Literature research office of the CPC central committee, 2013). This formulation revealed the significance of honest labor values and creative labor values. On May 1, 2015, Chairman Xi pointed out that society should establish the values that “laborers are supreme, laborers are equal, laborers are respectable, laborers are the most glorious, laborers are the most beautiful.” This expression reflected the significance of valuing equal labor, respecting labor, and loving labor (Xi, 2015). In 2016, in relevant symposiums, Chairman Xi emphasized, “humans are created by labor, and society is created by labor. There is no such thing as great or insignificant labor, and every profession is glorious…no matter what labor we undertake, we should do it, love it, and excel at it” (Xi, 2016). This expression showed value for equal labor and for loving labor. Through the analysis of Chinese academic world and official statements, we can conclude and extract that Chinese labor values mainly include values of honest labor, equal labor, treasuring labor, and distributing labor according to work.

Based on these, this study analyzed Chinese scholars’ and Chinese government’s formulations about labor education, extracting five dimensions of Chinese students’ labor values: honest labor values, equal labor values, treasuring labor values, loving labor values, and values of distribution according to work. According to the five dimensions and expert judgment, this study will construct relevant items of each dimension. This study chooses expert judgment for the construction of relevant items for two reasons: first, objects of this study are high-grade in primary school, but the concept of labor values is abstract, making it difficult to obtain effective qualitative data through open questionnaires. Second, in China, the concept of labor values is based on national policy, so its scale should reflect policy requirements. For these reasons, we chose teachers for the forefront discussions of relevant professional domains to construct specific projects under each dimension.

Meanwhile, to ensure validity of our questionnaire, this study used various calibrations. Because there is currently no measuring tool of labor values, this study chose two kinds of negative emotions, depression and anxiety, and two kinds of positive emotions, life satisfaction and subjective well-being, to test the validity of the questionnaire. Relevant data have shown that labor values significantly predict individual mental health (Eskin, 2013), including anxiety and depression. Previous studies also indicated that personal values have closed relationships with life satisfaction. Positive values are significantly and positively correlated with individual positive emotion (Ryan and Suzanne, 2001). Therefore, it is reasonable to choose anxiety, depression, life satisfaction, and happiness as the variables of the cross-validity test. In addition, according to the discoveries of relevant studies, the best time period during which to shape individual values is early adolescence (Shao and Liu, 2005). In the Chinese education system, the high grades (4, 5, and 6) in primary school coincide with early adolescence. Thus, high-grade students in primary school are ideal research subjects for construction of the measuring tool of labor values.

## Materials and Methods

### Item Generation for Questionnaire Development

Five professionals were gathered to discuss the specific performance of five dimensions, three of them from three prime schools studying the ideological and moral education of students and two of them from one university focused on moral research. During the discussion process, we confirmed the concepts and measured content on five dimensions of labor values, combining the communist party’s policy on labor education with each expert’s specific frontline experience. The honest labor value refers to the general viewpoint on down-to-earth and honest work, whose specific content involves the value judgment of a relationship, such as deception and reward or honest work and wealth. The equal labor value refers to the non-discrimination value orientation of individuals toward different divisions of labor and different subjects of labor. The specific contents include division of labor and the principle of equality. The cherishing labor value is defined as the value orientation of respecting others’ efforts and cherishing their achievements; specific contents include diligence and thrift, daily consumption, and general attitude to the achievements of others. The loving labor value is defined as the positive emotion or attitude of the individual toward the labor process and the labor result; specific content includes emphasizing labor practice and enjoying the labor process. The division value according to labor refers to the individual’s basic sense of social equity and justice in the relationship between labor pay and labor reward. The specific content includes the value judgment of the relationship between labor pay and reward.

### Preliminary Preparation of the Labor Values Scale

Based on the preliminary data collection defining the concept and content of each value dimension and referring to the compilation methods of some values scales, 24 items were developed, including three items on honest labor value (e.g., Everyone should gain wealth by honest labor), six items on equal status of labor value (e.g., There should be no nobility in a job), four items on cherishing labor value (e.g., Every grain must pass through labor, and it is shameful to waste), six items on loving labor value (i.e., I am happy in the process of labor), and five items on equal labor value (e.g., No pains, no gains). A 5-point Likert-type scale was adopted (1 = totally disagree, 5 = totally agree) with five reverse scoring items.

### Cognitive Interviews

After completing the preparation of the questionnaire project, we once again asked three experts from primary and secondary schools to evaluate the expression of the question, so as to ensure that the expression of the project content conforms to the reading comprehension 4–6 level of pupils.

### Measure

#### The Scale of Depression, Anxiety, and Stress

This scale was designed by Lovibond and Lovibond (1995) and was adapted into a Chinese version by Gong et al. (2010). There are 21 items, including three dimensions of anxiety, depression, and stress. In the present study, only two dimensions of anxiety and depression were used. A 4-point Likert-type scale was adopted (1 = no, 2 = sometimes, 3 = often, 4 = always). In this study, the Cronbach’s alpha coefficient was 0.832 for depression and 0.784 for anxiety.

#### Subjective Well-Being Scale

The scale was compiled by Lyubomirsky and Lepper (1999), consisting of four items, such as “In general, do you think you are living a happy life?” Each item was scored on a 6-point Likert-type scale (1 = very unhappy, 6 = very happy). The Cronbach’s alpha coefficient of this scale was 0.70.

#### Satisfaction With Life Scale

The scale was designed by Diener et al. (1985), consisting of five items, such as “I am satisfied with my life now.” A 5-point Likert-type scale was adopted. This scale has been confirmed to have a high reliability among Chinese participants (Kong et al., 2012; Kong and You, 2013; Xiang et al., 2020). In the present study, the Cronbach’s alpha coefficient of this scale was 0.833.

### Data Collection and Description

In the present study, cluster sampling was adopted, and all participants were selected from three primary schools in Shenzhen. Shenzhen is one of the most developed coastal cities in China, and all the students come from cities. Participants took the time between classes, gathered in the classroom, and completed the questionnaire under the guidance of the principal investigator. The professional personnel who were employed to carry out the test received systematic training. A total of 1,127 questionnaires were collected, among which 32 were forced to give up because they did too slowly and exceeded the prescribed time. In addition, 17 subjects filled in the questionnaires with obvious rules, so they were deleted. Finally, 1,078 valid questionnaires were collected, 553 males and 525 females, mean age = 11.38 ± 0.92 years; age range = 10–13 years. This study was approved by the ethics committee of the author’s research institute. We also obtained consent from the guardians of minor students. And all students completed the test on a voluntary basis.

### Data Analysis

All data were processed by SPSS20.0 and Amose7.0. First, we used critical ratio method and correlation analysis method to analyze the item. Secondly, we divided the data into sample1 (539 people) and sample2 (539 people) according to the random number method, conducted exploratory factor analysis with sample1, and eliminated the item according to the relevant standards. Third, we used Amose to conduct confirmatory factor analysis and explore whether there is a potential single factor or second-order factor. Fourthly, the internal reliability and validity of each dimension of the scale were calculated, and the external relevance validity was calculated. In addition, to evaluate the model rationality, comparative fit index (CFI), non-normal fit index (NNFI), root mean square error of approximation (RMSEA), and standardized root mean square residual (SRMR) indicators were used. According to the conventions of previous studies, the model fit was considered acceptable when CFI and NNFI ≥ 0.90, RMSEA ≤ 0.10, and SRMR ≤ 0.05 (Hu and Bentler, 1999).

## Results and Analyses

### Item Analysis

First, the critical ratio method and correlation analysis method were used to calculate the item differentiation. Subjects were arranged according to the order of the scale’s total scores, and the highest and lowest 27% of participants were divided into the high score group and the low score group. Independent sample *T*-test found that all high and low score groups had significant differences in each item (*p* < 0.001). At the same time, Pearson correlation analysis revealed that the correlation value between each item and the total score of the scale was between 0.40 and 0.46, which was also extremely significant (*p* < 0.001). It indicates that the discrimination of each item is relatively good.

### Exploratory Factor Analysis

For exploratory factor analysis, half of the samples were selected randomly and used. The results showed that the Barlett test chi-square was 3,810.953 (*p* < 0.001, df = 105), which indicated the possibility of shared factors among the items and was suitable for exploratory factor analysis. We used the principal component analysis and oblique rotation method to extract factors, taking an eigenvalue greater than 1 as the basic principle of factor extraction, and carried out exploratory factor analysis on 24 items. We then deleted the item according to the following criteria: (1) the commonality of each item is less than 0.30; (2) the maximum factor load is less than 0.40; (3) the cross loading is greater than 0.15. After several exploratory factor analyses, a total of 15 items with five factors was finally obtained, and the total variance could be explained as 59.756% (Table 1). Factor 1 is the honest labor value, including three items, mainly involving the attitude and value judgment of honest labor. Factor 2 is the equal labor value, which consists of three items, mainly involving attitudes and views on labor of different natures. Factor 3 is the cherishing labor value, including three items, mainly involving the value and cherishing labor value. Factor 4 is the loving labor value, which consists of three items, mainly involving the attitude toward the process of labor participation itself. Factor 5 is the distribution value according to work, including three items, mainly involving the concept of fair and reasonable labor and pay. Based on the results of the above exploratory factor analysis, it can be concluded that the structure of the labor values questionnaire in this study is reasonable.

**TABLE 1:** Factor normalized load estimation results basing on exploratory factor analysis.

Items	Honest labor value	Items	Equal Labor value	Items	Cherishing labor value	Items	Loving labor value	Items	Division value according to labor
S1	0.67	S2	0.64	S5	0.61	S11	0.41	S14	0.55
S4	0.34	S12	0.40	S8	0.40	S6	0.43	S17	0.59
S9	0.57	S3	0.48	S22	0.55	S13	0.54	S23	0.60

### Confirmatory Factor Analysis

In confirmatory factor analysis, cross validation is one of the most commonly used methods to calculate external validity. According to the results of exploratory factor analysis, we established a research model with five factors using the other half of the sample, each of which corresponds to different items, each with independent errors and unique factors. The maximum likelihood (ML) was used to fit the model.

The fitting indices of the five-factor model are as follows: χ^2^ = 404.19, df = 80, *p* < 0.001, χ^2^/df = 5.052, root mean square residual (RMR) = 0.056, goodness of fit index (GFI) = 0.952, adjusted goodness of fit (AGFI) = 0.927, Tucker–Lewis index (TLI) = 0.886, RMSEA = 0.061, 90% CI: 0.055–0.067, and the fitting index of the model has reached a good standard (Sharma et al., 2005). In addition, the load of each observed variable on the latent variable and the load on the error or unique factor are also important criteria for measuring the quality of the measurement model. Table 2 lists the item loads for each factor.

**TABLE 2:** Factor normalized load estimation results basing on confirmatory factor analysis.

Items	Honest labor value	Items	Equal labor value	Items	Cherishing labor value	Items	Loving labor value	Items	Division value according to labor
S1	0.78	S2	0.82	S5	0.53	S11	0.38	S14	0.58
S4	0.31	S12	0.49	S8	0.38	S6	0.73	S17	0.52
S9	0.68	S3	0.41	S22	0.45	S13	0.81	S23	0.55

**FIGURE 1:**
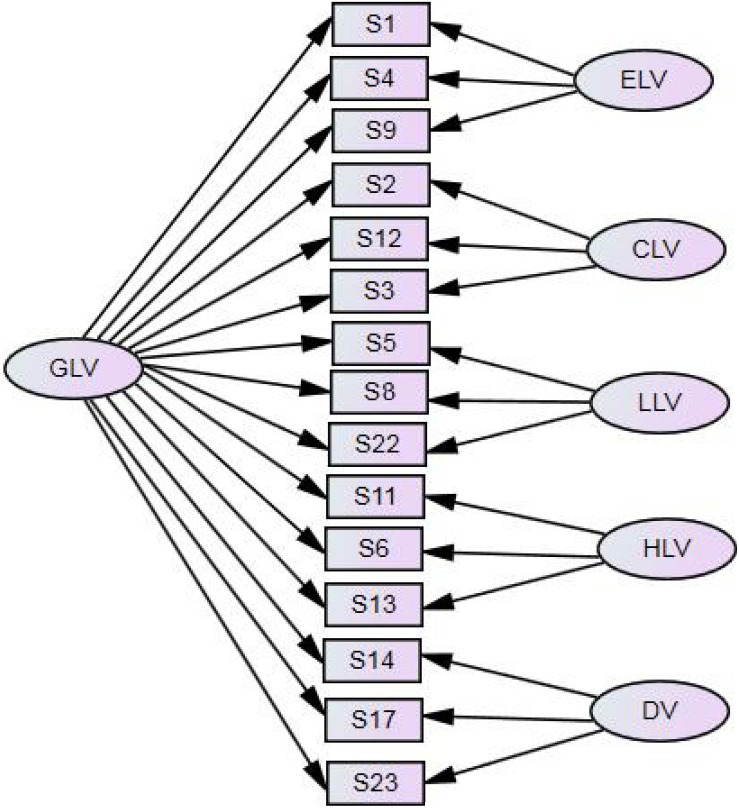
The bifactor model diagram. GLV, general labor value; ELV, equal labor value; CLV, cherishing labor value; LLV, love labor value; HLV, honest labor value.

In order to further explore whether the five factors belong to a higher order factor, we construct a latent variable based on the five first-order factors. It is found that the fitting result of the model is not ideal and does not reach the target of model fitting. The specific results are as follows: χ^2^ = 577.973, *df* = 85, *p* < 0.001, χ^2^/*df* = 6.800, RMR = 0.065, GFI = 0.868, AGFI = 0.892, TLI = 0.837, RMSEA = 0.093, 90% CI: 0.068–0.079. In addition, according to previous studies, any multidimensional scale should try to establish bifactor models (Gu et al., 2014). The results show that the fitting results of the bifactor models are ideal, and the specific parameters are as follows: χ^2^ = 215.602, *df* = 65, *p* < 0.001, χ^2^/*df* = 3.317, RMR = 0.035, GFI = 0.974, AGFI = 0.952, TLI = 0.935, RMSEA = 0.046, 90% CI: 0.040–0.053. This result shows that the structure of the questionnaire conforms to the bifactor model, which includes the general factors of labor values and five special factors (Figure 1).

### The Reliability Analysis in Each Factor

The results show that the Cronbach’s alpha values of each questionnaire dimension of labor values are as follows: the honest labor value was 0.67, the equal labor value was 0.71, the cherishing labor value was 0.65, the loving labor value was 0.75, the distribution according to work value was 0.76, and the total value of the questionnaire was 0.81. In summary, the internal consistency coefficient of the whole questionnaire and the sub-questionnaire conforms to the requirements.

### Criterion-Related Validity Analysis

To date, few studies have established measuring tools for labor values, and the labor value that we construct is mainly positive value tendency. Therefore, we selected two typical negative emotions (depression and anxiety) and two positive emotional states (life satisfaction and subjective well-being) as the criterion validity. The results showed that each dimension was significantly negatively correlated with negative emotional depression and anxiety and positively correlated with positive state subjective well-being, life satisfaction, and positive trait self-efficacy, showing that the questionnaire has good criterion validity (Table 3).

**TABLE 3:** Each dimension is related to positive and negative emotions and states.

Dimensions	Depression	Anxiety	Subjective well-being	Life satisfaction
Honest labor value	−0.244***	−0.183***	0.278***	0.304***
Equal labor value	−0.159***	−0.100**	0.202***	0.232***
Cherishing labor value	−0.237***	−0.179***	0.317***	0.280***
Loving labor value	−0.327***	−0.239***	0.384***	0.454***
Division value according to labor	−0.277***	−0.185***	0.351***	0.401***
Total points	−0.346***	−0.247***	0.427***	0.394***

***p < 0.001.

### Measurement Invariance Across Genders

To test the configural invariance model across sex, a multigroup CFA was performed. The result indicated that the model had a good index (RMSEA = 0.046, CFI = 0.91), and that all factor loading values were significant (*p* < 0.001). These results found no gender difference for this measurement tool.

## Discussion

According to procedure from Boateng et al. (2018), this study constructed the scale of labor values based on Chinese fundamental realities and relevant policy, by taking a novel sample population of high-grade (4, 5, and 6) primary school students. This scale filled the gap in Chinese labor values measurement and contributes important practical and theoretical value for relevant studies abroad.

Based on practical considerations, this study took the Chinese academic world’s theoretical and political analysis of labor values as the logical start and constructed dimensions of the scale. The original intention of this method was based on the particularity of labor values under the Chinese political system. During the new period of Jinping Xi, the Communist Party of China takes labor values as important components of the socialist core value system (Hao, 2014). In a way, this determines that what Chinese labor values aim to achieve is to establish a socialist outlook on labor value (Xu, 2019). The socialist labor value outlook has impressed the mark of a specific political culture at the beginning of its theoretical discussion. Therefore, the logical starting point of the questionnaire’s theoretical construction should follow suit. Only in this way can we make the created values measurement fit China’s national condition. As such, after constructing the theory, we discussed each dimension’s concept definition and measurement by expert judgment instead of *via* an open-ended questionnaire. The reason for using expert judgment is that teachers on the forefront, as well as experts, can more accurately grasp labor education and the policy orientation of labor value cultivation. Because of this, each dimension’s concept definition and measurement can more precisely reflect national conditions and educational reality. An additional reason for using expert judgment was that our objects were pupils. Because of age development characteristics, we could not provide valuable reference material for measuring labor values, an abstract concept, *via* an open-ended questionnaire.

After preparing items and finishing the measurement, we first conducted exploratory factor analysis. According to commonalities, the max load, the crossing load, and some other indices, we canceled some unqualified items. The results showed that five dimensions, represented by 15 items, can reflect that the population variance explained almost 60%. In the subsequent confirmatory factor analysis results, according to each question and their latent variable’s loading value, it showed that the test items under each dimension could better represent the corresponding dimension. At the same time, GFI, AGFI, RMSEA, normal fit index (NFI), CFI, and some other indices all reached statistical standard. It showed that data fit well. Each partial questionnaire has good construct validity. In further examination of related validity, according to the coefficient of association between positive–negative emotion and each dimension of labor values, we can judge that the questionnaire of labor values also has good construct validity. The reliability test also showed that the internal consistency reliability of the questionnaire reaches statistical standard. The data analysis revealed this study’s labor values scale to be in accordance with scientific standard.

In summary, based on labor values’ theoretical and political analysis, this study discussed the construction of labor values *via* an empirical approach. Results showed that various indices of the scale all arrived within the statistical standard. However, it is noteworthy that the five values’ dimensions were put forward based on a theoretical analysis. Whether or not there are unknown dimensions of labor values warrants further study. At the same time, this study performed its sample analysis on high-grade primary school students, whether it is suitable for middle school students remains unknown. In addition, China is a country with a large population. On the whole, the sample size used by this research only accounts for a small part of the population, and it is mainly concentrated in coastal cities. Therefore, it remains to be further studied to what extent this research can objectively reflect the values of the student labor force in other regions of China’s mainland. Finally, due to China’s special cultural and political system, the applicability of the labor value scale needs to be cautious.

## Data Availability Statement

The raw data supporting the conclusions of this article will be made available by the authors, without undue reservation.

## Ethics Statement

The studies involving human participants were reviewed and approved by Ethics Committee of Hunan Normal University. Written informed consent to participate in this study was provided by the participants’ legal guardian/next of kin.

## Author Contributions

All authors listed have made a substantial, direct and intellectual contribution to the work, and approved it for publication.

## Conflict of Interest

The authors declare that the research was conducted in the absence of any commercial or financial relationships that could be construed as a potential conflict of interest.
